# Age-Related Macular Degeneration and the Incidence of Cardiovascular Disease: A Systematic Review and Meta-Analysis

**DOI:** 10.1371/journal.pone.0089600

**Published:** 2014-03-28

**Authors:** Juan Wu, Miki Uchino, Srinivas M. Sastry, Debra A. Schaumberg

**Affiliations:** 1 Department of Nutrition, Harvard School of Public Health, Boston, Massachusetts, United States of America; 2 Department of Ophthalmology, Keio University School of Medicine, Tokyo, Japan; 3 Suburban Hospital, Bethesda, Maryland, United States of America; 4 Division of Preventive Medicine, Brigham and Women's Hospital, Harvard Medical School, Boston, Massachusetts, United States of America; 5 Moran Center for Translational Medicine, John A. Moran Eye Center, Department of Ophthalmology & Visual Sciences, University of Utah School of Medicine, Salt Lake City, Utah, United States of America; 6 Department of Epidemiology, Harvard School of Public Health, Boston, Massachusetts, United States of America; 7 Schepens Eye Research Institute, Boston, Massachusetts, United States of America; University of Utah, United States of America

## Abstract

**Importance:**

Research has indicated some shared pathogenic mechanisms between age-related macular degeneration (AMD) and cardiovascular disease (CVD). However, results from prior epidemiologic studies have been inconsistent as to whether AMD is predictive of future CVD risk.

**Objective:**

To systematically review population-based cohort studies of the association between AMD and risk of total CVD and CVD subtypes, coronary heart disease (CHD) and stroke.

**Data Sources:**

A systematic search of the PubMed and EMBASE databases and reference lists of key retrieved articles up to December 20, 2012 without language restriction.

**Data Extraction:**

Two reviewers independently extracted data on baseline AMD status, risk estimates of CVD and methods used to assess AMD and CVD. We pooled relative risks using random or fixed effects models as appropriate.

**Results:**

Thirteen cohort studies (8 prospective and 5 retrospective studies) with a total of 1,593,390 participants with 155,500 CVD events (92,039 stroke and 62,737 CHD) were included in this meta-analysis. Among all studies, early AMD was associated with a 15% (95% CI, 1.08–1.22) increased risk of total CVD. The relative risk was similar but not significant for late AMD (RR, 1.17; 95% CI, 0.98–1.40). In analyses restricted to the subset of prospective studies, the risk associated with early AMD did not appreciably change; however, there was a marked 66% (95% CI, 1.31–2.10) increased risk of CVD among those with late AMD.

**Conclusion:**

Whereas the results from all cohort studies suggest that both early and late AMD are predictive of a small increase in risk of future CVD, subgroup analyses limited to prospective studies demonstrate a markedly increased risk of CVD among people with late AMD. Retrospective studies using healthcare databases may have inherent methodological limitations that obscure such association. Additional prospective studies are needed to further elucidate the associations between AMD and specific CVD outcomes.

## Introduction

Age-related macular degeneration (AMD) is the leading cause of blindness among older persons in the US and other developed countries. About 9 million adults aged 40 years or older in the US are affected by different forms and severity of AMD [1], and the number of persons having AMD is projected to double by 2020 owing to the rapidly aging population [1]. AMD is categorized into early- and late-stage AMD according to AMD signs and severity. Most visual loss occurs in late AMD, which manifest itself in two forms, neovascular AMD and central geographic atrophy [2].

Cardiovascular disease (CVD), which includes both coronary heart disease (CHD) and cerebrovascular disease (stroke), remains the leading cause of deaths in both men and women globally. In the US, 1 in 3 adults have one or more types of CVD [3]. Epidemiologic studies have identified a number of CVD risk factors, among which age [4], ethnicity [5], obesity [6], cigarette smoking [7], hypertension [8] and high cholesterol [9] have also been associated with increased risk of AMD. In addition to traditional CVD risk factors, recent studies have suggested that C-reactive protein (CRP), a marker of systematic inflammation and independent risk factor for CVD, is associated with AMD as well [10], [11]. Furthermore, common genetic variants (apolipoprotein E [12], [13], complement factor H [14], [15]) have been linked to both CVD and AMD, though not necessarily in the same manner. In some studies [8], [16], [17], CVD has been shown as an independent risk factor for AMD, and are consistent with the hypothesis that AMD and CVD may share common underlying pathogenic mechanisms [18].

However, it remains unclear if in some instances AMD may manifest first and thus whether AMD might serve as an indicator for future risk of CVD. Prior studies of this issue were inconsistent and the work was underpowered to evaluate the potential differential associations of early versus late AMD with CVD. The goal of this study was to carry out a systematic review and meta-analysis of population-based cohort studies to estimate the risk of total CVD, as well as CVD subtypes, CHD and stroke, associated with early AMD and late AMD.

## Methods

### Search Strategy and Eligibility Criteria

One investigator (JW) conducted a systematic literature search of the PubMed and EMBASE databases and the reference list of retrieved articles up to December 20, 2012. The following terms were used for the search: (“macular degeneration” or “maculopathy”) and (“cardiovascular diseases” or “heart disease” or “stroke” or “coronary disease”) (detailed search terms available in **Table S1**). No language restriction was applied. Two investigators (JW, MU) independently screened all the retrieved articles for inclusion and exclusion. We conducted this systematic review according to the guidelines of Meta-analysis of Observation Studies in Epidemiology (MOOSE) (**Table S2**) [19].

Studies were eligible for inclusion if they met the following criteria: 1) the study design was a population-based cohort study; 2) the baseline exposure was AMD; 3) The outcome was CVD (not CVD risk factors or biomarkers); 4) The study provided a multivariate-adjusted effect estimate (hazard ratio, odds ratio or relative risk) for the association of AMD with CVD. We defined early AMD according to the Wisconsin age-related maculopathy grading system (WARMGS) [20], as the presence of soft drusen alone (distinct or indistinct), pigmentary abnormalities alone, or a combination of these in the absence of late AMD signs. Late AMD included the presence of neovascular AMD or central geographic atrophy. For studies that divided AMD into neo- and non-neovascular AMD types, we treated the non-neovascular AMD as early AMD in the primary meta-analysis, because early AMD accounted for at least 88% of the non-neovasular AMD cases [1]. We defined CVD as a composite outcome including CHD, CHD deaths, stroke, stroke deaths, and other atherosclerotic events. When multiple studies were published from the same cohort, we included only the most up-to-date analyses in which the highest number of CVD outcomes had accrued. A study that assessed two different CVD subtypes was counted as two studies in analysis unless it also assessed total CVD.

### Data Extraction

Two investigators (JW, MU) independently extracted data using a standardized electronic form. Discrepancies were resolved by discussion with the senior investigator (DAS). For each included study, we documented the title, study name, authors, publication year, country, study design, sample size, race/ethnicity, mean age and range, mean body mass index (BMI), proportion of men, mean duration of follow-up, the number of subjects with AMD at baseline (early and late AMD, respectively), AMD ascertainment method, the number of CVD cases, CVD ascertainment method, covariates adjusted for, risk estimates including 95% confidence intervals. Two types of AMD assessment method were used by included studies: in the prospective studies, retinal photographs were taken and were graded according to WARMGS [20]; whereas in retrospective studies, AMD was identified from claims data according to International Classification of Diseases codes, Ninth Revision (ICD-9). Methods of CVD ascertainment also differed between prospective and retrospective studies, with the former based on self-report followed by medical record review and the latter based on claims data and ICD-9 codes. If more than one multivariable-adjusted relative risk (RR) was provided, we extracted the effect estimate with the greatest adjustment for potential confounders.

### Statistical Analysis

We log transformed RRs and 95% CIs to obtain the corresponding standard errors for the *β*–coefficients according to the Greenland's formula [21]. In the Rotterdam Eye Study where RRs were reported across graded stage of AMD (stage 0–4, with stage 1–3 being early AMD and 4 being late AMD) [22], we used generalized least-squares trend estimation (GLST) [23] method to estimate the log-linear dose-response slope within stage 0 to 3 to approximate the overall RR associated with early AMD. The RR provided for the fourth stage of AMD would then represent the association of late AMD with CVD. The imputed RRs from the Rotterdam Eye Study were then pooled with RRs from other studies to derive an overall risk estimate using random-effects models, which took into account both the within-study and between-study variability. The *I^2^* statistic was used to evaluate the heterogeneity between studies [24]. Fixed-effects models were only used when the *I^2^* was below 30%. Of note, fixed-effects and random-effects model yields exactly the same results when *I^2^* = 0% (indicating no between-study heterogeneity).

To explore heterogeneity among studies, we used STATA METAREG to examine if the risk estimates were significantly different according to certain study-level characteristics, including study design (prospective or retrospective studies), publication year, study location, race/ethnicity (black/white, mostly white, Asian and mixed), mean age, proportion of men, mean duration of follow-up. In subgroup analyses, we further tested for the presence of effect modification by method of AMD assessment, CVD ascertainment and BMI.

We performed the primary meta-analyses separately for early and late AMD in relation to total CVD. Subgroup analyses were conducted to examine the associations of each with stroke and CHD. We also conducted sensitivity analyses by excluding retrospective studies from both primary and, if possible, subgroup analyses. Publication bias was assessed by visual inspection of funnel plots as well as by Begg's and Egger's tests [25]. All tests were two-sided, and P<0.05 was considered as statistically significant. We used STATA version 11 (StataCorp, College Station, Texas) to perform all the statistical analyses.

## Results

### Study population

**Figure 1:**
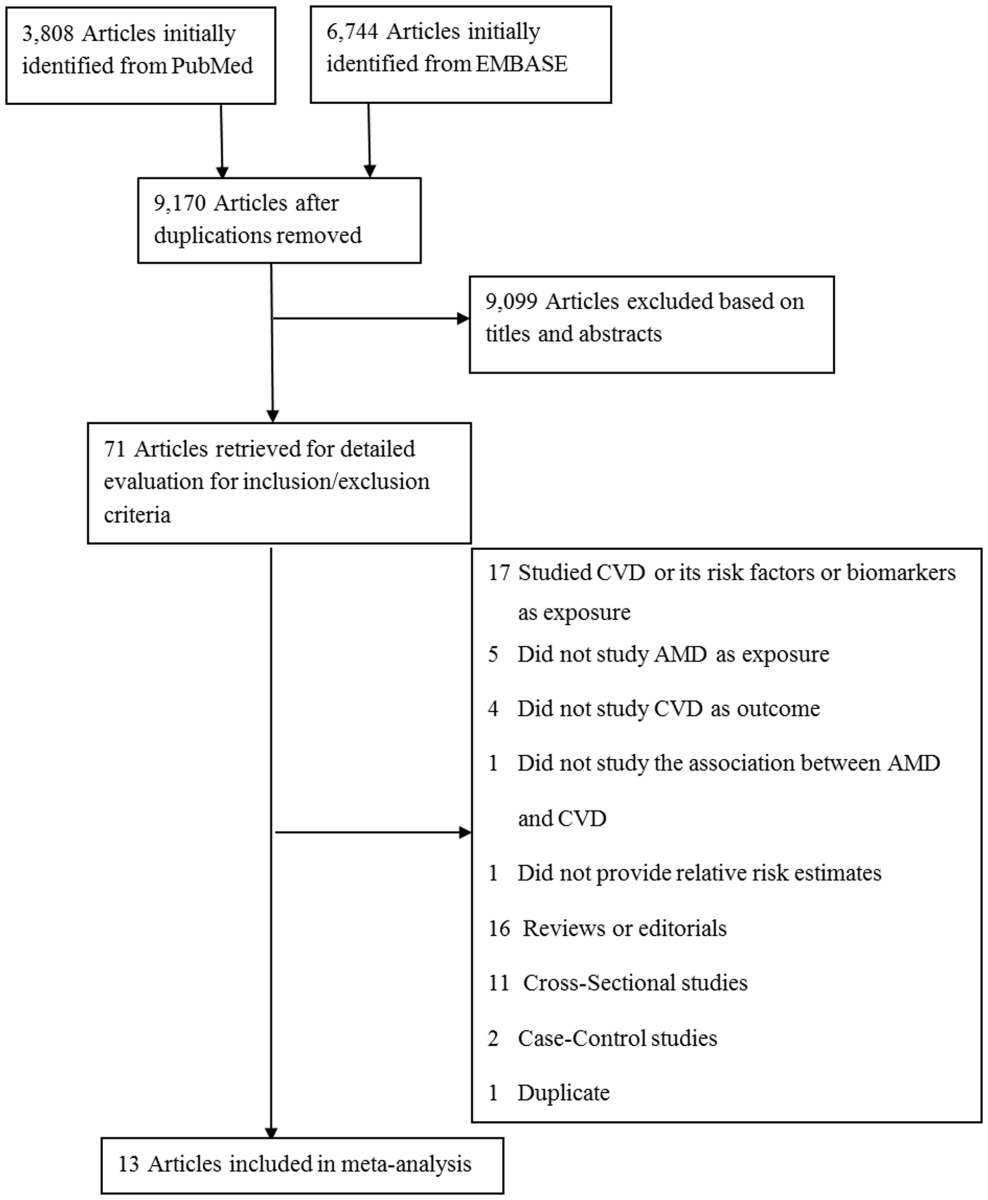
Flowchart of literature search for meta-analysis. None.

Of the 13 unique studies that met the inclusion criteria, 5 were retrospective cohort studies [26]–[30] using healthcare claims databases and 8 were prospective cohort studies [22], [31]–[37] (**Figure 1**). The 13 studies included 2 from Asia, 1 from Europe, 1 from Australia and 9 from North America. In sum, 155,500 CVD cases (including 92,039 strokes and 62,737 CHDs) were identified among 1,593,390 participants. The mean age of the study populations at baseline ranged from 59.8 to 78.4 years, average BMI ranged from 26.0 to 28.8 kg/m^2^ and the proportion of men varied from 34.4% to 60.3%. The mean duration of follow-up varied from 2.0 to 13.6 years, with longer follow-up generally seen in prospective studies (**Table 1**).

**Table 1:** Characteristics of the identified 13 cohort studies that evaluated the associations of AMD with CVD.

Author	Study name	Subjects,	Ethnicity	Mean age	Mean BMI,	Men,	Mean	AMD,	Late	AMD assessment
(year)	(country)	No.		(range), y	kg/m^2^	%	follow-up, y	No.	AMD, %	
**Stroke**										
Ikram et al	ARIC	12,216	White/black	59.9	28.4	44.2	13.0	591	2.5	Fundus Photography WARMGS
2012	USA			(45.0-64.0)						
Wieberdink et al 2011	Rotterdam Netherlands	6,207	White	67.6	26.0	40.4	13.6	2,259	4.1	Fundus Photography Stage grading^a^
				(61.7-74.8)						
Hu et al	LHID	1,254	Asian	62.7	-	60.3	5.0	209	100	Claims database
2010	Taiwan			(69.0-97.0)						ICD-9
Sun et al	CHS	2,228	White/Black	78.4	27.0	38.9	6.0	379	7.7	Fundus Photography WARMGS
2009	USA			(69.0-97.0)						
Tan et al	BMES Australia	2,853	White	65.1	26.1	41.3	11.0	181	28.2	Fundus Photography WARMGS
2008				(49.0-97.0)						
Liao et al	Medicare USA	1,303,186	White/Black	75.0	-	40.0	2.0	137,838	19.7	Claims database
2008				(≥65.0)						ICD-9
Nguyen-Khoa et al	LabRx	27,411	-	75.7	-	41.1	2.8	7,203	100	Claims database
2008	USA			(50.0-95.0)						ICD-9
Knudtson et al	BDES	4,926	White	62.0	28.8	43.9	13.2	988	8.0	Fundus Photography WARMGS
2006	USA			(43.0-84.0)						
**CHD**										
Fernandez	MESA	6,233	Mixed	62.0	28.3	47.6	5.4	895	3.0	Fundus Photography WARMGS
2012	USA			(45.0-84.0)						
Golan	MHS	68,218	White	68.5	-	41.5	11.0	6,546	-	Claims database
2011	Israel			(≥65.0)						ICD-9
Sun et al	CHS	1,786	White/black	78.2	27.0	34.3	6.0	302	8.3	Fundus Photography WARMGS
2009	USA			(69.0-97.0)						
Nguyen-Khoa et al 2008	LabRx	27,411	-	75.7	-	41.1	2.8	7,203	100	Claims database
	USA			(50.0-95.0)						ICD-9
Wong et al	ARIC	11,414	White/black	59.8	28.5	42.3	8.0	555	2.7	Fundus Photography WARMGS
2007	USA			(49.0-73.0)						
Duan et al	Medicare	1,445,677	White/black	76.0	-	40.0	2.0	157,579	19.6	Claims database
2007	USA			(≥65.0)						ICD-9
Knudtson et al	BDES	4,926	White	62.0	28.8	43.9	13.2	988	8.0	Fundus Photography WARMGS
2006	USA			(43.0-84.0)						
**CVD**										
Fernandez	MESA	6,233	Mixed	62.0	28.3	48.0	5.4	895	3.0	Fundus Photography WARMGS
2012	USA			(45.0-84.0)						
Tan et al	BMES	2,853	White	65.1	26.1	41.3	11.0	181	28.2	Fundus Photography WARMGS
2008	Australia			(49.0-97.0)						
AREDS research	AREDS	4,753	Mostly	69.0	27.5	44.2	6.5	4,753	20.1	Fundus Photography
group 2004	USA		white	(55.0-81.0)						AREDS

Author	Outcome,	Outcome ascertainment	Adjustment for covariates	Study design
(year)	No.			
**Stroke**				
Ikram et al	619	Self-report confirmed by	Age, sex, race, field center, mean ABP, antihypertensive medication, fasting glucose,	Prospective
2012		medical record	cholesterol, HDLC, triglyceride, BMI, atrial fibrillation, white blood cell count,	
Wieberdink et al	726	Database linkage and	Age, sex, diabetes, SBP, antihypertensive medication, smoking, total cholesterol,	Prospective
2011		medical record review	HDLC, BMI, carotid artery plaques, CRP, and alcohol	
Hu et al	142	Claims database	Age, sex, hypertension, diabetes, CHD, hyperlipidemia, renal disease, monthly	Retrospective
2010		ICD-9	income, urbanization level, and geographic region	
Sun et al	198	Self-report confirmed by	Age, sex, ethnicity, SBP and DBP, hypertension, fasting glucose, diabetes,	Prospective
2009		medical record	triglyceride, smoking (pack-years and current), LDLC, CRP	
Tan et al	99	Linkage with national death	Age, sex, hypertension, diabetes, smoking, and BMI	Prospective
2008		database		
Liao et al	89,501	Medicare claims database	Age, sex, race, hypertension, and diabetes	Retrospective
2008		ICD-9		
Nguyen-Khoa et al	549	Claims database	Angina, cardiac arrhythmia, Charlson comorbidity score, congestive heart failure,	Retrospective
2008		ICD-9	diabetes, heart disease, history of acute MI, history of CVA, hyperlipidemia,	
			hypertension, and other cerebrovascular disease (AMD and non-AMD were matched	
			on age, sex, geography and duration of enrollment)	
Knudtson et al	205	Death Certificate	Age, sex, BMI, BMI squared, proteinuria, SBP, and diabetes	Prospective
2006		ICD-9		
**CHD**				
Fernandez et al	278	Self-report confirmed by	Race, hypertension, smoking, enrollment site, CRP, education, diabetes, BMI, serum	Prospective
2012		medical record	cholesterol, and LDLC	
Golan	3,156	Claims database	Age, sex, diabetes and hypertension	Retrospective
2011		ICD-9		
Sun et al	303	Self-report confirmed by	Age, sex, ethnicity, SBP and DBP, hypertension, fasting glucose, diabetes,	Prospective
2009		medical record	triglyceride, smoking (pack-years and current), LDLC, CRP	
Nguyen-Khoa et al	584	Claims database	Angina, cardiac arrhythmia, Charlson comorbidities score, congestive heart failure,	Retrospective
2008		ICD-9	diabetes, heart disease, history of acute MI, history of CVA, hyperlipidemia,	
			hypertension, and other cerebrovascular disease (AMD and non-AMD were matched	
			on age, sex, geography and duration of enrollment)	
Wong et al	922	Self-report confirmed by	Age, sex, race, field center, education, SBP, DBP, diabetes, total cholesterol,	Prospective
2007		medical record	triglyceride, glucose, HDLC, smoking (pack-years), and alcohol	
Duan et al	56,611	Medicare claims database	Age, sex, race, hypertension and diabetes	Retrospective
2007		ICD-9		
Knudtson et al	883	Death Certificate ICD-9	Age, sex, BMI, BMI squared, ratio of total cholesterol to HDLC, smoking, pulse,	Prospective
2006			diabetes, sedentary lifestyle, education, SBP	
**CVD**				
Fernandez et al	384	Self-report confirmed by	Race, hypertension, smoking, enrollment site, CRP, education, diabetes, BMI, serum	Prospective
2012		medical record	cholesterol, and LDLC	
Tan et al	183	Linkage with national death	Age, sex, hypertension, diabetes, smoking, and BMI	Prospective
2008		database		
AREDS research	534	Medical record and death	Age, sex, race, education, smoking, BMI, diabetes, angina, cancer and hypertension	Prospective
group 2004		certificate		

Abbreviations: ARIC, Atherosclerosis Risk in Communities Study; MESA, Multi-Ethnic Study of Atherosclerosis; CHS, Cardiovascular Health Study; BMES, Blue Mountains Eye Study; BDES, Beaver Dam Eye Study; LHID, Longitudinal Health Insurance Database; LabRx, Ingenix LabRx Database; Medicare, Medicare beneficiaries databases; MHS, Maccabi Healthcare Services; AREDS: Age-Related Eye Disease Study; WARMGS, Wisconsin age-related maculopathy grading system; ICD-9, International Classification of Disease codes, Ninth Revision; ABP, Arterial blood pressure; HDLC, high-density lipoprotein cholesterol; LDLC, low-density lipoprotein cholesterol; BMI, body mass index; SBP, systolic blood pressure; DBP, diastolic blood pressure; MI, myocardial infarction; CVA, cerebrovascular accident; CRP, C-reactive protein.

a AMD was graded by stage 0–4 (0, no AMD), a grading scheme similar to the WARMGS.

### Early AMD with CVD

**Figure 2:**
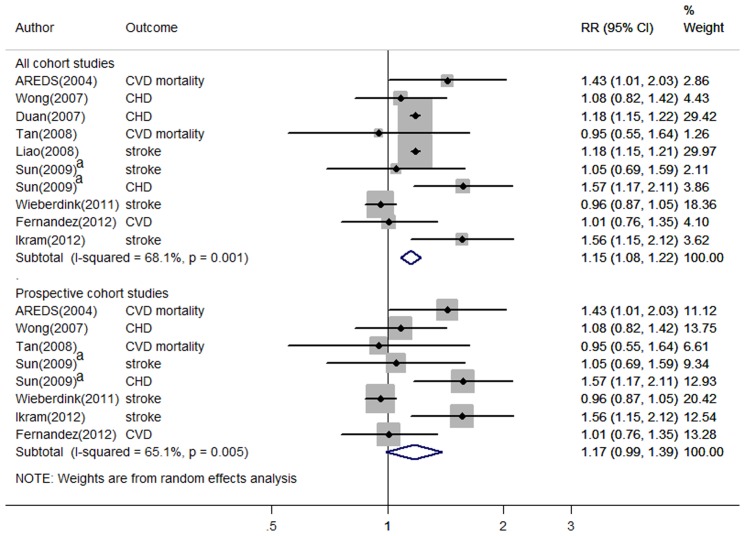
Relative risks of total CVD associated with early AMD. Top panel represents forest plot among all relevant cohort studies. Bottom panel represents forest plot among all relevant prospective cohort studies. Open data markers indicate the adjusted RR of CVD comparing persons with early AMD to persons without early AMD. The size of the data markers represents the weight of the study. Diamond indicates the pooled RR. ^a^ The study evaluated CHD and stroke separately without providing an overall RR for total CVD.

**Figure 3:**
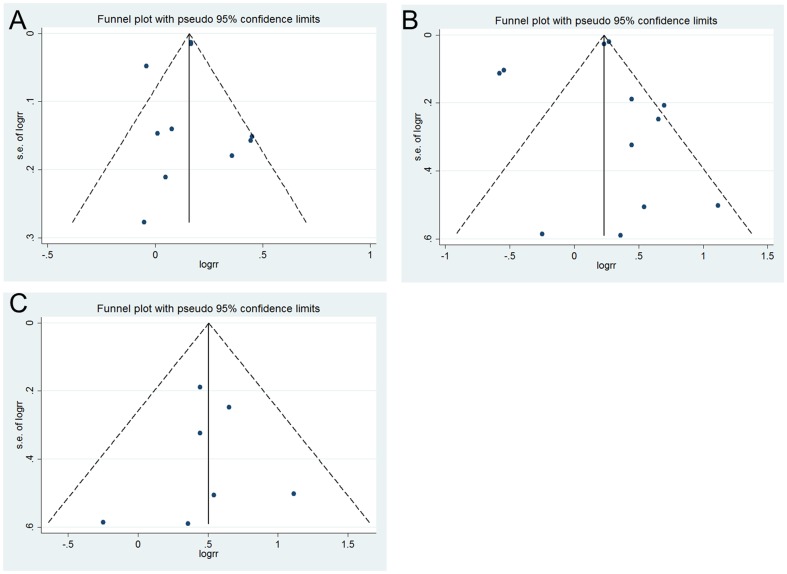
Funnel plots for the detection of public bias among studies that evaluated the associations of AMD with total CVD. The vertical line across the X-axis is the log of the pooled RR. Dotted lines are pseudo 95% CIs corresponding to a given standard error. Each dot represents one identified study. A symmetric distribution of dots around the vertical line indicates minimal publication bias. Figure 3A, early AMD with CVD. Begg's test, p = 0.93; Egger's test, p = 0.81; Figure 3B, late AMD with CVD. Begg's test, p = 0.73; Egger's test, p = 0.62; Figure 3C, late AMD with CVD among prospective studies. Begg's test, p = 0.76; Egger's test, p = 0.86.

Across 10 studies with information on early AMD [22], [26], [27], [32]–[37], early AMD was significantly associated with a 15% (95% CI, 1.08–1.22) increased incidence of total CVD (**Figure 2**). However, substantial between-study heterogeneity was observed (*I^2^* = 68%, p = 0.001). Study design did not explain the heterogeneity (p for interaction, 0.90) Begg's (p = 0.93) and Egger's tests (p = 0.81) for publication bias were not significant but a visual inspection of the funnel plot revealed slight asymmetry, suggesting possible publication bias (**Figure 3**). Restricting analysis to prospective studies did not appreciably change the pooled RR or reduce between-study heterogeneity (**Figure 2**).

With respect to stroke, the pooled RR associated with early AMD was 1.11 (95% CI, 0.94–1.32) across 5 studies. After excluding the one retrospective study, the RR was slightly attenuated to 1.07 (95% CI, 0.79–1.45) (**Table 2**). As the stratified analysis by stroke subtypes showed, there appeared to be a slightly stronger association between AMD and risk of intracerebral hemorrhage (RR, 1.29; 95% CI, 1.00–1.68) as compared to cerebral infarction (RR, 1.14; 95% CI, 0.96–1.35) (**Table 2**). With respect to CHD, early AMD was associated with a significant 19% (95% CI, 1.05–1.35) increased risk of CHD across 4 studies. After excluding the only retrospective study, the RR was slightly higher but no longer significant (RR, 1.21; 95% CI, 0.93–1.57) (**Table 2**).

**Table 2:** The pooled relative risks for CVD subtypes in relation to early and late AMD.

Exposure	Outcome	Study type	Data points, No.	Summary RR (95% CI)	P for heterogeneity	*I^2^, %*
**Early AMD**						
	Stroke	All	5	1.11 (0.94, 1.32)	<0.001	83
	Stroke	Prospective	4	1.07 (0.79, 1.45)	0.02	71
	*ICH	All	3	1.29 (1.00, 1.68)	0.14	49
	*CI	All	3	1.14 (0.96, 1.35)	0.01	81
	CHD	All	4	1.19 (1.05, 1.35)	0.20	35
	CHD	Prospective	3	1.21 (0.93, 1.57)	0.10	56
**Late AMD**						
	Stroke	All	6	1.15 (0.74, 1.78)	<0.001	92
	Stroke	Prospective	3	1.43 (1.02, 2.00)	0.27	24
	CHD	All	5	1.12 (0.64, 1.96)	<0.001	93
	CHD	Prospective	3	1.64 (0.75, 3.61)	0.21	36

* ICH, intracerebral hemorrhage; *CI, cerebral infarction.

### Late AMD with CVD

**Figure 4:**
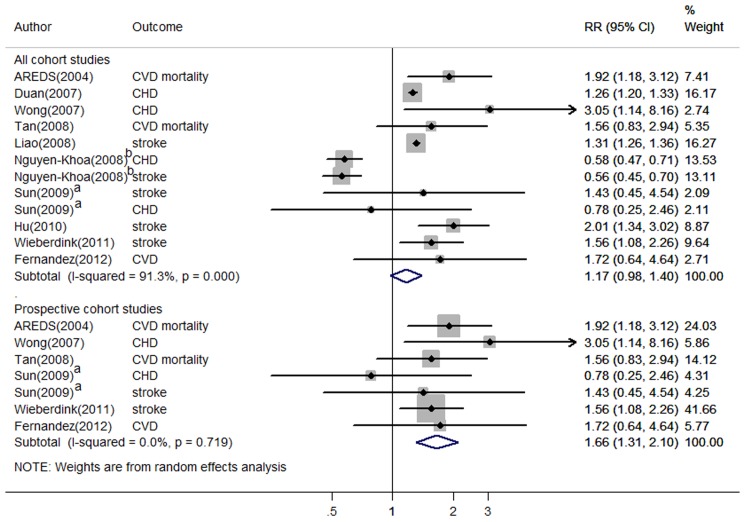
Relative risks of total CVD associated with late AMD. See footnote of Figure 2. ^a,b^ The studies evaluated CHD and stroke separately without providing overall RRs for total CVD.

Across 12 studies with information on late AMD [22], [26]–[29], [32]–[34], [36], [37], the pooled RR for total CVD associated with late AMD was 1.17 (95% CI, 0.98–1.40) (**Figure 4**). Substantial between-study heterogeneity was evident (*I^2^* = 91%, p<.001). Which CVD outcomes were assessed did not contribute to the heterogeneity (p for interaction, 0.53). We found that mean age at baseline significantly modified the association between late AMD and incident CVD (p for interaction, 0.01). Higher risk was seen in studies with mean participant age <75 years, in which the pooled RR was 1.80 (95% CI, 1.46–2.23), whereas in studies with mean participant age ≥75 years, the RR was 0.90 (95% CI, 0.72–1.13). Elevated risk of CVD among those with late AMD was also only seen in studies with duration of follow-up greater than 5 years (>5 y: RR, 1.74, 95% CI, 1.42–2.41 versus ≤5 y: RR, 0.89, 95% CI, 0.71–1.12; p for interaction, 0.02). Both Begg's (p = 0.73) and Egger's (p = 0.62) tests were not significant but a visual inspection of funnel plot indicated potential publication bias (**Figure 3**). To further explore between-study heterogeneity, we separately analyzed the associations among the prospective and retrospective studies. In the subgroup of 7 prospective studies, there was a strong and significant association between late AMD and total CVD with a RR of 1.66 (95% CI, 1.31–2.10) (**Figure 4**). Little heterogeneity was found among prospective studies (*I^2^* = 0%, p = 0.72). In contrast, the pooled analysis among all retrospective studies revealed substantial heterogeneity (RR, 0.99; 95% CI, 0.80–1.23; *I^2^* = 97%), suggesting that it was mainly the retrospective studies driving the overall between-study heterogeneity. Among prospective studies, the same pattern of effect modification by baseline mean age persisted, although the test for interaction was not significant likely due to a reduction in power for this test (data not shown). Duration of follow-up was no longer a significant effect modifier, but all prospective studies had at least 5 years of mean follow-up. Funnel plot was fairly symmetric and tests for publication bias were not significant (**Figure 3**).

In subgroup analysis for stroke, the pooled RR associated with late AMD was 1.15 (95% CI, 0.74–1.78) based on 6 studies. When the retrospective studies were excluded, the RR for stroke associated with late AMD was statistically significant at 1.43 (95% CI, 1.02–2.00) (**Table 2**). We did not evaluate the associations of late AMD with stroke subtypes as only 2 studies [22], [27] evaluated this association. The 2 studies exhibited opposite direction of associations regarding cerebral infarction but the same positive associations with intracerebral hemorrhage. Such differential association between late AMD and different stroke types is consistent with what we observed for early AMD. With respect to CHD, the association between late AMD and CHD was not significant (RR, 1.12; 95% CI, 0.64–1.96) in the primary analysis, though the RR was elevated when the two retrospective studies were excluded (RR, 1.64; 95% CI, 0.75–3.61) (**Table 2**).

## Discussion

In the present systematic review, both early and late AMD were associated with a modest increased incidence of CVD when data from both prospective and retrospective cohort studies were summarized. Analyses restricted to the subset of prospective cohort studies, however, identified a stronger 66% increased risk of CVD among persons with late AMD. There was also a trend towards increased risk of CHD and stroke among those with early AMD and late AMD but findings were limited by a small number of studies.

Subgroup analyses revealed that the association between late AMD and risk of CVD was only evident among populations with a mean age <75 years. This observation might be explained by an increase with aging in the prevalence of other CVD risk factors and/or in the absolute risk of CVD. Alternatively, people with a strong genetic predisposition to late AMD may have an earlier age of onset, and may be the group more likely to have increased risk of CVD due to genetic or other factors. Such a finding could also be explained by selective mortality bias in that the removal of susceptible persons results in falsely attenuated associations in the older population. It is of interest that this finding is consistent with results in the Blue Mountains Eye Study, in which late AMD was significantly associated cardiovascular mortality among participants younger than 75 years old, but not in participants 75 years or older [38].

A recent meta-analysis by Chakravarthy et al [39] has investigated if some CVD risk factors and CVD itself are predictive of the development of late AMD. In that study, CVD was found to be only weakly and non-significantly associated with future risk of late AMD among prospective cohort studies (RR, 1.22; 95% CI, 0.92–1.63). Therefore, whether CVD could be used as a risk indicator for late AMD remains inconclusive. Our study, on the other hand, demonstrated that in some cases when late AMD manifests first, it might serve as a useful indicator of a higher risk for future CVD events. Moreover, whereas Chakravarthy et al. restricted their analysis to associations of CVD with late AMD, we included early AMD cases and found a modest association between early AMD and risk of future CVD. Thus, our work and that published by Chakravarthy et al collectively support the shared antecedents hypothesis between AMD and CVD. In our study, the increasing magnitude of association with future CVD from early to late AMD is biologically plausible, and, clinically, implies that a cardiovascular risk assessment for AMD patients starting at the early stage of the disease may be desirable for early detection of underlying CVD. Of note, the association between AMD and future CVD has been adjusted for shared risk factors. Therefore, the association may reflect some other aspects of shared pathogenic processes, such as those due to diet and genetics. If applied clinically, the positive predictive value of AMD for CVD might be even higher, as it reflects a composite pathogenic process of clinically observable as well as unknown or unmeasurable underlying risk factors. However, more research is needed to understand the possible utility of CVD screening among patients with AMD.

Several mechanisms for observed associations of AMD with CVD have been proposed. Chronic inflammation is a plausible biologic mechanism for both AMD and CVD. The fact that complement cascade activation plays a role in drusen formation [14], [40] and the identification of some genetic variants of complement proteins as strong risk factors for AMD lends support to a role of local inflammation in the pathogenesis of AMD. However, although earlier epidemiologic studies suggested a positive link between common AMD-associated variants in complement factor H and CHD, a recent meta-analysis with a large sample size and high statistical power did not confirm such association, nor was any association found between AMD-associated CFH variants and a wide range of CHD risk factors or biomarkers except triglycerides [41]. These data suggest that the relationship between AMD and CVD is probably more complex than a single shared risk factor or even a single shared biological pathway, and more work is clearly needed. Atherosclerosis may also play a pathogenic role in the development of AMD due to its effect on choroidal circulation and lipid deposition at Bruch's membrane [42]. Oxidized lipids would bind to macrophages, trigger downstream inflammatory cascade and result in atherosclerotic lesions [43]. All these suggest that AMD is a complex disorder stemming from interactions between inflammation, atherosclerosis and oxidative stress, pathogenic processes that are also related to CVD.

In interpreting this meta-analysis, some limitations should be noted. In primary analyses among all cohort studies, substantial heterogeneity was evident and hence the pooled RRs should be interpreted cautiously. We explored the sources of heterogeneity and identified that study design contributed most of the heterogeneity in the analysis of late AMD in relation to CVD. Retrospective cohort studies conducted based on healthcare claims databases were prone to a number of limitations. Residual and unmeasured confounding was one of them, as such studies often failed to account for confounding factors such as BMI, smoking, physical activity and diet, which are important risk factors for CVD. In addition, coding inaccuracies and lack of clinical validation may have led to misclassification of both AMD and CVD. These studies also had a shorter duration of follow-up, and thus there may have been insufficient time for an increased risk of CVD to become manifest. As a result, such studies may have been biased in an unpredictable direction, as demonstrated by the substantial heterogeneity when pooling RRs only among retrospective cohort studies. In contrast, there was no significant between-study heterogeneity among prospective studies of late AMD. This is expected, as prospective studies generally had better adjustment for a wide range of potentially confounding factors, longer duration of follow-up (ranging from 5 to 13 y), as well as standardized procedures for AMD and CVD ascertainment. Based on these observations, we think that the pooled risk estimates from the subset of prospective cohort studies are likely to be the most reliable and informative. However, restricting to prospective studies did not effectively reduce the heterogeneity in the analysis of early AMD with CVD. This may be due to the inherent heterogeneity of early AMD with respect to varying stage and severity and to the likely misclassification as a result of detection bias – most early AMD are asymptomatic. Another limitation of this meta-analysis is related to the different components of total CVD, which may have different associations with AMD. However, since the test for heterogeneity was nonsignificant, pooling together all CVD events would greatly increase the statistical power. A minor limitation was introduced by the imputed RR by GLST in the Rotterdam Eye Study: the resulting overall RR should only be interpreted as the RR of stroke for per one level increase in AMD stage. However, since the HR was null across each stage of early AMD in this study, we argue that GLST-generated RR, although not ideal, could approximate the true HR. With only 13 studies included for meta-analysis, our findings may be prone to publication bias. Finally, our meta-analysis had limited ability to perform reliable subgroup analyses in relation to each CVD subtype. Prospective cohort studies conducted in the younger elderly with large sample sizes, long duration of follow-up and standardized protocol in AMD assessments and CVD ascertainments are warranted to further clarify the issues above.

Our meta-analysis also has several strengths. We built our analyses on population-based cohort studies and excluded case-control and cross-sectional analyses, which greatly reduced the likelihood of selection bias and recall bias and established temporality. We have included population-based cohort studies with wide geographic locations and varied population characteristics, which increased the generalizability of our results. In addition, we have investigated separately the associations between early and late AMD with CVD. The consistent findings and a “dose-response” relationship, as shown in the prospective studies, not only increased the validity of our findings but also provided additional clinically relevant information, as early AMD is the predominant type of AMD in the older persons. Owing to the relatively low prevalence of late AMD (1.5% among Americans 40 years of age or older [1]), most prospective cohort studies were not able or underpowered to assess its association with CVD, let alone with CVD subtypes, which necessitates pooled analysis with enhanced statistical power, such as we conducted.

In conclusion, our meta-analysis based on best-available evidence from prospective cohort studies indicates that early and late AMD are independent predictors of future CVD, and the association is much stronger for late AMD. Although retrospective studies using healthcare databases had much higher statistical power, the inherent methodological limitations may prohibit making reliable inferences about associations. Additional prospective studies are still warranted to provide further insights into the associations of AMD with CVD and individual cardiovascular endpoints. Clinically, our study implies that an increase in cross-disciplinary awareness among ophthalmologists, optometrists, cardiologists, and other physicians of the link between these two diseases could have beneficial health consequences for patients.

## Supporting Information

Table S1 **Meta-analysis search terms for PubMed and EMBASE.**
(DOCX)

Table S2 **MOOSE Checklist.**
(DOCX)

Checklist S1 **PRISMA Checklist.**
(DOC)
