# Correction: Age-Related Macular Degeneration and the Incidence of Cardiovascular Disease: A Systematic Review and Meta-Analysis

**DOI:** 10.1371/journal.pone.0102216

**Published:** 2014-07-01

**Authors:** The PLOS ONE Staff

Table 1 is formatted incorrectly. The publisher apologizes for this error. The authors have provided a corrected version here.

**Table 1:** Characteristics of the identified 13 cohort studies that evaluated the associations of AMD with CVD.

Author (year)	Study name (country)	Subjects, No.	Ethnicity	Mean age (range), y	Mean BMI, kg/m^2^	Men, %	Mean follow-up, y	AMD, No.	Late AMD, %	AMD assessment
**Stroke**										
Ikram et al 2012	ARIC USA	12,216	White/black	59.9 (45.0-64.0)	28.4	44.2	13.0	591	2.5	Fundus Photography WARMGS
Wieberdink et al 2011	Rotterdam Netherlands	6,207	White	67.6 (61.7-74.8)	26.0	40.4	13.6	2,259	4.1	Fundus Photography Stage grading a
Hu et al 2010	LHID Taiwan	1,254	Asian	62.7 (69.0-97.0)	-	60.3	5.0	209	100	Claims database ICD-9
Sun et al 2009	CHS USA	2,228	White/Black	78.4 (69.0-97.0)	27.0	38.9	6.0	379	7.7	Fundus Photography WARMGS
Tan et al 2008	BMES Australia	2,853	White	65.1 (49.0-97.0)	26.1	41.3	11.0	181	28.2	Fundus Photography WARMGS
Liao et al 2008	Medicare USA	1,303,186	White/Black	75.0 (≥65.0)	-	40.0	2.0	137,838	19.7	Claims database ICD-9
Nguyen-Khoa et al 2008	LabRx USA	27,411	-	75.7 (50.0-95.0)	-	41.1	2.8	7,203	100	Claims database ICD-9
Knudtson et al 2006	BDES USA	4,926	White	62.0 (43.0-84.0)	28.8	43.9	13.2	988	8.0	Fundus Photography WARMGS
**CHD**										
Fernandez 2012	MESA USA	6,233	Mixed	62.0 (45.0-84.0)	28.3	47.6	5.4	895	3.0	Fundus Photography WARMGS
Golan 2011	MHS Israel	68,218	White	68.5 (≥65.0)	-	41.5	11.0	6,546	-	Claims database ICD-9
Sun et al 2009	CHS USA	1,786	White/black	78.2 (69.0-97.0)	27.0	34.3	6.0	302	8.3	Fundus Photography WARMGS
Nguyen-Khoa et al 2008	LabRx USA	27,411	-	75.7 (50.0-95.0)	-	41.1	2.8	7,203	100	Claims database ICD-9
Wong et al 2007	ARIC USA	11,414	White/black	59.8 (49.0-73.0)	28.5	42.3	8.0	555	2.7	Fundus Photography WARMGS
Duan et al 2007	Medicare USA	1,445,677	White/black	76.0 (≥65.0)	-	40.0	2.0	157,579	19.6	Claims database ICD-9
Knudtson et al 2006	BDES USA	4,926	White	62.0 (43.0-84.0)	28.8	43.9	13.2	988	8.0	Fundus Photography WARMGS
**CVD**										
Fernandez 2012	MESA USA	6,233	Mixed	62.0 (45.0-84.0)	28.3	48.0	5.4	895	3.0	Fundus Photography WARMGS
Tan et al 2008	BMES Australia	2,853	White	65.1 (49.0-97.0)	26.1	41.3	11.0	181	28.2	Fundus Photography WARMGS
AREDS research group 2004	AREDS USA	4,753	Mostly white	69.0 (55.0-81.0)	27.5	44.2	6.5	4,753	20.1	Fundus Photography AREDS

Author (year)	Outcome, No.	Outcome ascertainment	Adjustment for covariates	Study design
**Stroke**				
Ikram et al 2012	619	Self-report confirmed by medical record	Age, sex, race, field center, mean ABP, antihypertensive medication, fasting glucose, cholesterol, HDLC, triglyceride, BMI, atrial fibrillation, white blood cell count	Prospective
Wieberdink et al 2011	726	Database linkage and medical record review	Age, sex, diabetes, SBP, antihypertensive medication, smoking, total cholesterol, HDLC, BMI, carotid artery plaques, CRP, and alcohol	Prospective
Hu et al 2010	142	Claims database ICD-9	Age, sex, hypertension, diabetes, CHD, hyperlipidemia, renal disease, monthly income, urbanization level, and geographic region	Retrospective
Sun et al 2009	198	Self-report confirmed by medical record	Age, sex, ethnicity, SBP and DBP, hypertension, fasting glucose, diabetes, triglyceride, smoking (pack-years and current), LDLC, CRP	Prospective
Tan et al 2008	99	Linkage with national death database	Age, sex, hypertension, diabetes, smoking, and BMI	Prospective
Liao et al 2008	89,501	Medicare claims database ICD-9	Age, sex, race, hypertension, and diabetes	Retrospective
Nguyen-Khoa et al 2008	549	Claims database ICD-9	Angina, cardiac arrhythmia, Charlson comorbidity score, congestive heart failure, diabetes, heart disease, history of acute MI, history of CVA, hyperlipidemia, hypertension, and other cerebrovascular disease (AMD and non-AMD were matched on age, sex, geography and duration of enrollment)	Retrospective
Knudtson et al 2006	205	Death Certificate ICD-9	Age, sex, BMI, BMI squared, proteinuria, SBP, and diabetes	Prospective
**CHD**				
Fernandez et al 2012	278	Self-report confirmed by medical record	Race, hypertension, smoking, enrollment site, CRP, education, diabetes, BMI, serum cholesterol, and LDLC	Prospective
Golan 2011	3,156	Claims database ICD-9	Age, sex, diabetes and hypertension	Retrospective
Sun et al 2009	303	Self-report confirmed by medical record	Age, sex, ethnicity, SBP and DBP, hypertension, fasting glucose, diabetes, triglyceride, smoking (pack-years and current), LDLC, CRP	Prospective
Nguyen-Khoa et al 2008	584	Claims database ICD-9	Angina, cardiac arrhythmia, Charlson comorbidities score, congestive heart failure, diabetes, heart disease, history of acute MI, history of CVA, hyperlipidemia, hypertension, and other cerebrovascular disease (AMD and non-AMD were matched on age, sex, geography and duration of enrollment)	Retrospective
Wong et al 2007	922	Self-report confirmed by medical record	Age, sex, race, field center, education, SBP, DBP, diabetes, total cholesterol, triglyceride, glucose, HDLC, smoking (pack-years), and alcohol	Prospective
Duan et al 2007	56,611	Medicare claims database ICD-9	Age, sex, race, hypertension and diabetes	Retrospective
Knudtson et al 2006	883	Death Certificate ICD-9	Age, sex, BMI, BMI squared, ratio of total cholesterol to HDLC, smoking, pulse, diabetes, sedentary lifestyle, education, SBP	Prospective
**CVD**				
Fernandez et al 2012	384	Self-report confirmed by medical record	Race, hypertension, smoking, enrollment site, CRP, education, diabetes, BMI, serum cholesterol, and LDLC	Prospective
Tan et al 2008	183	Linkage with national death database	Age, sex, hypertension, diabetes, smoking, and BMI	Prospective
AREDS research group 2004	534	Medical record and death certificate	Age, sex, race, education, smoking, BMI, diabetes, angina, cancer and hypertension	Prospective

Abbreviations: ARIC, Atherosclerosis Risk in Communities Study; MESA, Multi-Ethnic Study of Atherosclerosis; CHS, Cardiovascular Health Study; BMES, Blue Mountains Eye Study; BDES, Beaver Dam Eye Study; LHID, Longitudinal Health Insurance Database; LabRx, Ingenix LabRx Database; Medicare, Medicare beneficiaries databases; MHS, Maccabi Healthcare Services; AREDS: Age-Related Eye Disease Study; WARMGS, Wisconsin age-related maculopathy grading system; ICD-9, International Classification of Disease codes, Ninth Revision; ABP, Arterial blood pressure; HDLC, high-density lipoprotein cholesterol; LDLC, low-density lipoprotein cholesterol; BMI, body mass index; SBP, systolic blood pressure; DBP, diastolic blood pressure; MI, myocardial infarction; CVA, cerebrovascular accident; CRP, C-reactive protein.

a AMD was graded by stage 0-4 (0, no AMD), a grading scheme similar to the WARMGS.
