# Supplementary material for: Age-Related Macular Degeneration and the Incidence of Cardiovascular Disease: A Systematic Review and Meta-Analysis
Source: PLoS One. 2014 Mar 28;9(3):e89600. doi: 10.1371/journal.pone.0089600 (PMC3969321; doi:10.1371/journal.pone.0089600)
Supplement: Table S1 — Meta-analysis search terms for PubMed and EMBASE. (DOCX) [file pone.0089600.s001.docx]

**Table S1. Meta-analysis search terms for PubMed and EMBASE.**

| **Search Site** | **Search Date** | **No. of Publications** | **Search Terms** |
| --- | --- | --- | --- |
| PubMed | 12/20/2012 | 3,808 | ("macular degeneration"[MeSH] OR "macular degeneration"[All Fields] OR maculopathy[All Fields]) AND ("cardiovascular diseases"[MeSH] OR "cardiovascular diseases"[All Fields] OR "cardiovascular disease"[All Fields] OR "coronary disease"[MeSH] OR "coronary disease"[All Fields] OR "coronary diseases"[All Fields] OR "myocardial infarction"[MeSH] OR "myocardial infarction"[All Fields] OR "myocardial infarctions"[All Fields] OR "heart diseases"[MeSH] OR "heart diseases"[All Fields] OR "heart disease"[All Fields] OR "stroke"[MeSH] OR "stroke"[All Fields] OR "strokes"[All Fields] OR "cerebrovascular accident"[All Fields] OR "cerebrovascular accidents"[All Fields]) |
| EMBASE | 12/20/2012 | 6,744 | 'macular degeneration'/exp OR 'macular degeneration' OR 'maculopathy'/exp OR maculopathy AND ('cardiovascular disease'/exp OR 'cardiovascular disease' OR ('heart'/exp AND 'diseases'/exp) OR 'stroke'/exp OR stroke OR 'cerebrovascular accident'/exp OR 'cerebrovascular accident' OR (myocardial AND ('infarction'/exp OR infarction)) OR (coronary AND ('disease'/exp OR disease))) |
