# Supplementary material for: Age-Related Macular Degeneration and the Incidence of Cardiovascular Disease: A Systematic Review and Meta-Analysis
Source: PLoS One. 2014 Mar 28;9(3):e89600. doi: 10.1371/journal.pone.0089600 (PMC3969321; doi:10.1371/journal.pone.0089600)
Supplement: Table S2 — MOOSE Checklist. (DOCX) [file pone.0089600.s002.docx]

**Table S2. MOOSE Checklist.**

| **Criteria** | **Way of handling** |
| --- | --- |
| **Reporting of background** |  |
| Problem definition | Research has indicated some shared pathogenic mechanisms between age-related macular degeneration (AMD) and cardiovascular disease (CVD). However, results from prior epidemiologic studies have been inconsistent as to whether AMD is predictive of future CVD risk. |
| Hypothesis statement | AMD might be predictive of future CVD risk |
| Description of study outcomes | Incident CVD and CVD subtypes (CHD and stroke) |
| Type of exposure or intervention used | Early and late AMD |
| Type of study designs used | Population-based cohort studies |
| Study population | Cohort studies with no restrictions |
| **Reporting of search strategy** |  |
| Qualifications of searchers | The credentials of the two investigators, JW and MU, are indicated in the author list. |
| Search strategy, including time period included in the synthesis and keywords | PubMed up to Dec, 20^th^, 2012  EMBASE up to Dec, 20^th^, 2012  Macular degeneration, maculopathy, cardiovascular disease, stroke, coronary heart disease |
| Databases and registries searched | PubMed and EMBASE |
| Search software used, name and version, including special features | We did not use any search software. Retrieved articles were merged by EndNote with duplications removed |
| Use of hand searching | We hand-searched the reference list of retrieved articles |
| List of citations located and those excluded, including justifications | Details of the literature search process are outlined in Figure 1. Full list of excluded articles are available upon request |
| Method of addressing articles published in languages other than English | We placed no restrictions on language. No articles in language other than English were observed. |
| Method of handling abstracts and unpublished studies | No unpublished studies were observed. |
| Description of any contact with authors | We contacted authors from one study where the RR was reported for one stage increase in AMD severity; however, we failed to hear back. |
| **Reporting of methods** |  |
| Description of relevance or appropriateness of studies assembled for assessing the hypothesis to be tested | We have pre-specified inclusion and exclusion criteria to ensure the appropriateness of studies for assessing the hypothesis |
| Rationale for the selection and coding of data | Results |
| Assessment of confounding | We evaluated the covariates adjusted for in each included study |
| Assessment of study quality, including blinding of quality assessors; stratification or regression on possible predictors of study results | We conducted sensitivity analysis by excluding retrospective studies |
| Assessment of heterogeneity | We assessed between-study heterogeneity by *I^2^* statistics and explored the sources of heterogeneity by meta-regression |
| Description of statistical methods in sufficient detail to be replicated | In the method section we described models used, meta-regression, GLST, and publication bias assessments |
| Provision of appropriate tables and graphics | We included in the manuscript 1 figure of flowchart, 2 figures of forest plots, 1 figure of publication bias, and 1 table summary of all study characteristics, as well as supplemental content. |
| **Reporting of results** |  |
| Graph summarizing individual study estimates and overall estimate | Figure 1 |
| Table giving descriptive information for each study included | Table 1 |
| Results of sensitivity testing | Results section in the manuscript |
| Indication of statistical uncertainty of findings | We reported all risk estimates with 95% confidence intervals |
| **Reporting of discussion** |  |
| Quantitative assessment of bias | We reported the results of sensitivity analyses. We also assessed publication bias according to Begg’s and Egger’s tests in addition to visual inspection of funnel plots |
| Justification for exclusion |  |
| Assessment of quality of included studies | We discussed the strengths of prospective cohort studies and the limitations of retrospective cohort studies |
| **Reporting of conclusions** |  |
| Consideration of alternative explanations for observed results | Discussion section in the manuscript |
| Generalization of the conclusions | AMD is of positive predictive value for future CVD |
| Guidelines for future research | We recommended future prospective cohort studies to be conducted in the younger population with longer duration of follow-up in order to further clarify the associations of AMD with CVD subtypes. |
| Disclosure of funding source | We reported the funding source to the journal |
